# The association of fat preference with eating behavior and sex: Turkish version of the Fat Preference Questionnaire^©^

**DOI:** 10.1002/fsn3.2237

**Published:** 2021-03-15

**Authors:** Binnur Okan Bakır, İrem Kaya Cebioğlu, Elif Günalan, Gözde Dumlu Bilgin

**Affiliations:** ^1^ Department of Nutrition and Dietetics Yeditepe University İstanbul Turkey; ^2^ Department of Nutrition and Dietetics İstanbul Health and Technology University Istanbul Turkey

**Keywords:** dietary restriction, fat preference, food choice, Turkish version

## Abstract

The fat content of food may play a role in food preferences. Increased fat intake has a role in elevated body weight. Firstly, we aimed to establish the Turkish version of the Fat Preference Questionnaire^©^ and secondly to evaluate the relevant factors with dietary fat preference including body mass index (BMI); sex; and subscales of the Three‐Factor Eating Questionnaire (TFEQ). The study was conducted with 261 participants among the academic staff of Yeditepe University. The Fat Preference Questionnaire^©^ and TFEQ were applied. After the validity and reliability of the Turkish version of the Fat Preference Questionnaire^©^, Pearson's correlation coefficients were calculated to reveal the relationship between the scores of the Fat Preference Questionnaire^©^, BMI, and the four subscales of TFEQ. Weakly or moderately correlated variables were selected to perform two sets of hierarchical regression analyses. Turkish version of the Fat Preference Questionnaire^©^ had statistically acceptable validity and reliability. Fat preference did not correlate with BMI (*p* > .05). Women showed a lower preference for high‐fat foods and a higher dietary fat restriction (*p* < .05). The two subscales of TFEQ, the Disinhibition of Eating Control and the Susceptibility to Hunger, contributed to explain the variances in fat preference and dietary fat restriction (Δ*R* ^2^ = .04, *p* < .05). Fat preference correlates with Disinhibition of Eating Control and Susceptibility to Hunger, while fat restriction correlates only with Disinhibition of Eating Control although none correlates with BMI. Turkish version of the Fat Preference Questionnaire^©^ is a valid instrument for further studies.

## Graphical abstract

The Turkish version of the Fat Preference Questionnaire^©^ is a statistically acceptable, valid, and reliable tool. Sex is a significant contributor on the preference of high‐fat foods. Fat preference correlates with Disinhibition of Eating Control and Susceptibility to Hunger. Fat restriction correlates with Disinhibition of Eating Control.

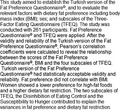

## 1 INTRODUCTION

Dietary fat intake in sufficient amounts is essential due to its role as being a major energy source and facilitating the absorption of fat‐soluble vitamins and carotenoids. In addition to its contribution to the maintenance of health if consumed in recommended amounts and types, it promotes a favorable flavor and provides intended texture while cooking (Vannice & Rasmussen, 2014). Thus, it is also considered that its textural and olfactory characteristics are effective on preference (Besnard, 2016). However, excessive amounts and unhealthy types may play a role in the increased risk of developing chronic diseases including cardiovascular diseases and obesity, which is related to increased morbidity and mortality (Freeland‐Graves & Nitzke, 2013; Jawaldeh & Al‐Jawaldeh, 2018; Macronutrients, 2005). It has been shown that increased fat intake contributes to increased calorie intake resulting in increased body weight regardless of the types consumed (Vannice & Rasmussen, 2014). Consistent with the global increase in obesity prevalence, Turkey was found to have “sharply increasing obesity rates” and overweight, and obesity was found to be much higher than the average for the World Health Organization (WHO) European Region (Jakab et al., 2014). On the other hand, there is a suggestion that diet‐induced obesity results in decreased sensitivity for fatty taste in rodents besides it is also a hypothesis in humans referring to an increased preference on high‐fat food compared with lean ones (Besnard, 2016; Douglas Braymer et al., 2017). Besides, sex is another suggested factor influencing food preference regarding fat content with numerous studies focused on women's high‐fat food preference (Aguiar‐Bloemer & Diez‐Garcia, 2018; Costanzo et al., 2017; Ledikwe et al., 2007; Van Langeveld et al., 2018) and a preference for more palatable food has been shown in female rodents (Freeman et al., 2020).

Moreover, the Three‐Factor Eating Questionnaire (TFEQ) was widely used for the determination of the factors underlying spontaneous food preferences (Karlsson et al., 2000; Kirac et al., 2015; Ledikwe et al., 2007; Stunkard & Messick, 1985). It evaluates eating behaviors and patterns of the individuals with and without obesity, and its subscales have been found associated with sex and obesity (Karlsson et al., 2000; Stunkard & Messick, 1985).

The Fat Preference Questionnaire^©^ is a valid 19‐item questionnaire developed by Ledikwe et al. (2007) enabling assessment of individual's fat preference on 19 sets of foods in which each set is composed of two or three forms of a specific food containing different amounts of fat (Ledikwe et al., 2007).

Initially, we aimed to establish Turkish validity and reliability of the Fat Preference Questionnaire^©^ to supply a tool adapted to the Turkish language and culture enabling assessment of spontaneous food preferences regarding fat content for further studies. After that, we attempted to evaluate the suggested relevant factors with dietary fat preference: BMI; sex; and subscales of the TFEQ including Cognitive Restraint of Eating; Disinhibition of Eating Control; Emotional Eating; and Susceptibility to Hunger.

## 2 METHODS

### 2.1 Participants

The sample size was calculated as 10‐fold of the total number of the items in the questionnaire (Akgül, 1997) with a number of 190 and an additional 10% rate of wastage, and we aimed to reach at a minimum of 210 participants from the academic staff of a university. The study was completed with 261 voluntary participants without any diseases requiring dietary restrictions between the ages of 18 and 65 years.

### 2.2 Data collection

Firstly, a written consent form was obtained from all participants. Sex, educational status, and body weight (kg) and height (cm) of the participants were recorded. BMI values were calculated and classified according to WHO criteria (World Health Organisation, 1995). The Fat Preference Questionnaire^©^ and Three‐Factor Eating Questionnaire (TFEQ) were applied via face‐to‐face interviews (83.5%) and online platforms (16.5%) with a formal announcement to the academic staff by the university.

### 2.3 Fat Preference Questionnaire^©^

The Fat Preference Questionnaire^©^ is a valid questionnaire developed by Ledikwe et al. (2007) with 19 food sets, and in each, there are 2–3 food alternatives that have different fat contents enabling assessment of individual's fat preference. In each set, firstly, it is asked if the individual has ever eaten that food; if yes, the respondent is asked which tastes better (TASTE score) and lastly which is consumed more often (FREQ score). DIFF scores are calculated by subtracting FREQ scores from TASTE scores (Ledikwe et al., 2007).

### 2.4 Turkish language adaptation

After receiving permission from the developers of the Fat Preference Questionnaire^©^, a translation to the Turkish language was applied with a procedure developed by Brislin (Brislin, 1986). Firstly, an informed researcher translated to Turkish from the original language and an uninformed lecturer from the department of foreign languages translated back to English. This procedure was repeated since there were no inconsistencies (Bracken & Barona, 1991).

### 2.5 Cultural adaptation

After the Turkish validation, a pilot study on 30 volunteers was applied to guarantee the clarity and appropriateness of the Turkish version of the questionnaire. For some food groups that were found difficult to understand due to cultural differences, some items were exampled (Brislin, 1986) to make them more apprehensible, and for some, we replaced some foods with the ones more commonly consumed and more familiar in the same food groups in Turkey. For the fifth item that was cream soups or clear soups and as the pilot group mentioned that they could not understand the types of clear soups, we exampled them with the frequently consumed ones in Turkish cuisine. Additionally, we made some adaptations for ice cream (4th item) for making it more clear as reduced fat ice cream alternative is not frequently available in Turkey, and we changed it as sorbet prepared with frozen fruits without milk or egg and exampled it to make it clearer. Also in the ninth item questioning the Scotch pancake consumption with or without butter/margarine, the Scotch pancake was replaced with similar pastry types that are consumed with butter/margarine in Turkish cuisine. Lastly, for vegetable consumption (15th item) the alternative for vegetable consumption with dip required examples for dip sauce.

The retest was conducted 2–3 weeks after the initial survey to establish reliability in which 120 of 261 participants completed the Fat Preference Questionnaire^©^ again.

### 2.6 Three‐Factor Eating Questionnaire

The Three‐Factor Eating Questionnaire (TFEQ) was developed by Stunkard and Messick (1985) (Stunkard & Messick, 1985) with 51 questions, and it was revised to an 18‐item version (Karlsson et al., 2000). In the revised version, another new subscale Emotional Eating was identified, and it was suggested that distinguishing disinhibition and hunger was impossible, thus formed it in one global factor entitled as uncontrolled eating, and they confirmed that Cognitive Restraint of Eating subscale was appropriate for remaining the same. Turkish validity and reliability of revised 18‐item TFEQ (Karlsson et al., 2000) were performed in 2015 (Kirac et al., 2015), which included the following factors: Cognitive Restraint of Eating; Disinhibition of Eating Control; Emotional Eating; and Susceptibility to Hunger.

### 2.7 Statistics

After the language and cultural adaptation of the Fat Preference Questionnaire**^©^**, Pearson's correlation coefficient was calculated to show the validity and reliability of the Turkish version. The Rasch analysis was used to determine the internal consistency of the non‐Likert scale. The Kaiser–Meyer–Olkin and Bartlett's tests were performed to assess the sample concordance for factor analyses. As data were found to be normally distributed, the test–retest reliability of the Fat Preference Questionnaire**^© ^**was evaluated by comparing Pearson's correlation coefficients of the scores from two administrations. All descriptive data for the Fat Preference Questionnaire**^©^** and eating inventory (TFEQ) were given for overall sample and according to the sex by mean ± SE. Additionally, the scores of the Fat Preference Questionnaire**^© ^**were displayed by median, ranges, and interquartile ranges. For further investigation of the possible differences between the sexes, the *t* test was conducted. Pearson's correlation coefficients were calculated to reveal the relationship between the scores of the Fat Preference Questionnaire**^©^**, BMI, and the four subscales of TFEQ. Based on the results of the correlations, the weakly or moderately correlated variables were selected to perform two sets of hierarchical regression analyses explaining the amount of variances in preference and restriction for high‐fat foods. As the Durbin–Watson test values were close to 2 and variance inflation factors of all variables were close to 1, it was assumed multicollinearity did not exist. All statistical analyses were performed using the R for the Windows software program and IBM SPSS Statistics for Windows (IBM Corp., Version 25.0). Statistical significance was accepted for *p* <.05.

## 3 RESULTS

The study was completed with 261 voluntary participants (67.3% women) with a mean age of 31.1 years and a mean BMI of 23.8 ± 0.3 kg/m^2^. 33.0% of the participants were overweight or obese with a BMI value of ≥25.0.

### 3.1 Turkish validity and reliability of the Fat Preference Questionnaire^©^

The internal consistency of the Turkish version of the Fat Preference Questionnaire^©^ was determined by Cronbach's alpha coefficient, which is found as 0.71 with the mean ± *SD* of 60.8 ± 18.5 and statistically accepted as internally reliable. The first and second results of the TASTE, FREQ, and DIFF scores were evaluated for test–retest reliability. Pearson's correlation coefficients were 0.75 (*p* <.001) for the TASTE scores; 0.83 (*p* <.001) for the FREQ scores; and 0.52 (*p* <.001) for the DIFF scores.

The Kaiser–Meyer–Olkin test was performed to control the sample concordance for factor analysis, and the Kaiser–Meyer–Olkin coefficient was found to be 0.71. Bartlett's test was performed for the data set's concordance for factor analysis (*χ* ^2^ = 490.230, *p* =.001). The main component analysis was used for factor analysis, and components with an eigenvalue greater than 1 were evaluated for determining factor number. Analysis of the 19 items showed that the questionnaire had five factors that have an eigenvalue greater than 1. Factor 1 with an eigenvalue of 3.58 explained 18.81% of the total variance; factor 2 with an eigenvalue of 1.66 explained 8.72%; factor 3 with an eigenvalue of 1.42 explained 7.48%; factor 4 with an eigenvalue of 1.29 explained 6.83%; and factor 5 with an eigenvalue of 1.06 explained 5.59% of the total variance. This 3‐factor structure has explained 52.71% of the total variance.

### 3.2 Eating inventory results

Kıraç et al. (Kirac et al., 2015) concluded that the Turkish version of the TFEQ scale evaluates Cognitive Restraint of Eating, Disinhibition of Eating Control and Emotional Eating, and also Susceptibility to Hunger. Thus, the TFEQ scale was evaluated in terms of 4 subscales. According to sex, women displayed higher scores than men for the Disinhibition of Eating Control and the Susceptibility to Hunger but lower scores for the Cognitive Restraint of Eating and the Emotional Eating. To reveal the significance of this difference, *t* test was conducted and eventually it was found that women displayed significantly higher Disinhibition of Eating Control than men (12.7 and 12.1, respectively, *p* <.05). Women had lower tendency to eat emotionally than men did (7.9 and 8.7, respectively; *p* <.05). Women showed lower Cognitive Restraint of Eating scores than men (15.8 versus 16.1, respectively), although this difference was statistically insignificant.

### 3.3 Fat preferences

Despite 60.8% of high‐fat options were selected as “tasting better” according to the overall TASTE scores, the FREQ scores that were lower with a mean of 49.2% of the high‐fat options were selected as being “eaten more often.” As a measure of dietary fat restriction of the participants, the DIFF scores were 11.9% calculated by subtracting the FREQ scores from the TASTE scores. Women reported lower TASTE scores (59.9%) compared with men (62.5%), but this difference was not significant. Similarly, high‐fat foods over low‐fat alternatives were selected as eaten significantly more often among men (55.4%) compared with women (46.2%) participants (*p* <.001). While the dietary fat restriction was 13.9% and 7.8% among women and men, respectively, the comparison analysis indicated that women eat significantly more restrictively compared with men regarding fat content of the diet (*p* <.01).

Table 1 shows the descriptive data; the Fat Preference Questionnaire^©^ scores; and the subscale scores of the eating inventory by sex.

**TABLE 1:** Sample characteristics, scores of eating inventory (TFEQ), and dietary fat preference

	Overall (*n* = 261)	Women (*n* = 176)	Men (*n* = 85)	Significance	
				*p* *	
Age (years)	31.1 ± 0.6	30.8 ± 0.6	31.7 ± 1.1	‐	
BMI (kg/m^2^)	23.8 ± 0.3	22.7 ± 0.3	26.2 ± 0.4	‐	
Subscale scores of TFEQ				
CR	15.9 ± 0.1	15.8 ± 0.2	16.1 ± 0.2	.290	
DE	12.5 ± 0.1	12.7 ± 0.1	12.1 ± 0.2	.014^a^	
EE	8.2 ± 0.2	7.9 ± 0.2	8.7 ± 0.3	.017^a^	
SH	11.7 ± 0.2	11.8 ± 0.2	11.6 ± 0.3	.709	
TASTE (%)					
Mean ± SE	60.8 ± 1.1	59.9 ± 1.4	62.5 ± 2.0	.280	
Median	63.2	63.2	63.2		
Ranges	2.6–100	10.5–100	2.6–95		
IR	47.4–73.7	47.2–73.7	52.6–78.4		
FREQ (%)					
Mean ± SE	49.2 ± 1.2	46.2 ± 1.4	55.4 ± 2.1	.000^b^	
Median	47.4	44.4	57.9		
Ranges	6.2–89.5	6.2–89.5	6.7–89.5		
IR	36.8–63.2	33.7–63.2	41.7–68.4		
DIFF (%)					
Mean ± SE	11.9 ± 0.9	13.9 ± 1.1	7.8 ± 1.5	.002^b^	
Median	10	10.5	5.3		
Ranges	−26.7–77	−26.7–77	−15.8–74		
IR	0–19.9	1.2–24.6	0–13.3		

Abbreviations: CR, cognitive restraint of eating; DE, disinhibition of eating control; EE, emotional eating; IR, interquartile range; SH, susceptibility to hunger.

^a^ Significant differences between women and men, *p* <.05.

^b^ Significant differences between women and men, *p* <.01.

* *p* value calculated with *t* test.

### 3.4 The relationship between fat preference, BMI, and eating inventory

The relationship between the Fat Preference Questionnaire^©^, BMI, and the subscales of eating inventory for the Turkish sample is given in Table 2. High‐fat foods that taste better (TASTE) were positively correlated with the foods eaten most often (FREQ) (*r* = .67, *p* <.001). The TASTE and the dietary fat restriction (DIFF) scores were found positively correlated indicating that as the taste perception of high‐fat foods increased, the restriction on diet increased as well. Unlike the TASTE scores, the FREQ scores were negatively correlated with the DIFF scores. As the frequency of consumption of high‐fat foods increased, the dietary restriction decreased. No relationship between the BMI and any scores of the Fat Preference Questionnaire^©^ was observed.

**TABLE 2:** Intercorrelations between the subscales of the Fat Preference Questionnaire^©^, BMI, and TFEQ

		TASTE	FREQ	DIFF	BMI	CR	DE	EE	SH
TASTE	R	‐							
FREQ	R	0.67^a^	‐						
DIFF	R	0.29^a^	−0.44^a^	‐					
BMI	R	0.04	0.02	−0.02	‐				
CR	R	0.06	0.08	−0.04	−0.03	‐			
DE	R	−0.08	−0.19^b^	0.14^c^	−0.10	0.10	‐		
EE	R	−0.10	−0.07	−0.08	−0.17^b^	0.26^a^	0.16^b^	‐	
SH	R	−0.09	−0.16^b^	0.13^c^	−0.15^c^	0.22^a^	0.35^a^	0.61^a^	‐

Abbreviations: CR, cognitive restraint of eating; DE, disinhibition of eating control; EE, emotional eating; SH, susceptibility to hunger.

^a^ *p* < .001.

^b^ *p* < .01.

^c^ *p* < .05.

In terms of the eating inventory subscales, BMI was negatively correlated with the Emotional Eating and the Susceptibility to Hunger (*r* = −.17, *p* <.01, and *r* = −.15, *p* <.05, respectively). The Disinhibition of Eating Control was negatively correlated with the FREQ (*r* = −.19, *p* <.01). There was a positive correlation with the DIFF scores (*r* = .14, *p* <.05). However, no relationship was found with the TASTE scores (*p* >.05). Besides, subscales of the Cognitive Restraint of Eating and the Emotional Eating did not correlate with any scores of the Fat Preference Questionnaire^©^. There was a negative correlation between the Susceptibility to Hunger and the FREQ (*r* = −.16, *p* <.01). Additionally, the Susceptibility to Hunger was positively correlated with the dietary restraint (DIFF) (*r* = .13, *p* <.05).

Hierarchical regression analyses were conducted with the FREQ and DIFF subscales of the Fat Preference Questionnaire^©^, which are entered as criterion variables to explain the relationship between “fat preference, and dietary fat restriction” and sex; the Disinhibition of Eating Control; and the Susceptibility to Hunger (Table 3).

**TABLE 3:** Hierarchical regression analyses on preference on high‐fat foods

FREQ	Step 1	*R* ^2^ = .05	ΔF_(1,259)_ = 13.1*	*β*	*p*
	Sex			−0.19	.01**
	Step 2	Δ*R* ^2^ = .04	ΔF_(2,257)_ = 5.3*		
	DE			−0.12	.06
	SH			−0.12	.07
DIFF	Step 1	*R* ^2^ = .04	ΔF_(1,259)_ = 10.1*		
	Sex			0.18	.004**
	Step 2	Δ*R* ^2^ = .02	ΔF_(2,257)_ = 2.9		
	DE			0.08	.21
	SH			0.10	.14

Abbreviations: DE, disinhibition of eating control; SH, susceptibility to hunger.

* *p* <.001.

** *p* <.01.

The preference for high‐fat foods (FREQ) was observed to be associated with sex, which explains almost 5% of the variances in the scores of FREQ. This result indicates that women (who had a normal BMI) preferred low‐fat foods. At the second step, as the Disinhibition of Eating Control and the Susceptibility of hunger were added to the model, the association between the criterion and predictor variables significantly increased by 4% (*p* <.01), although these were not significant contributors.

According to the second hierarchical regression, sex was a higher contributor to the model as it explains that women restricted their diets more. At the second step, even though adding the Disinhibition of Eating Control and the Susceptibility to Hunger to the model slightly increased the association to 6%, this contribution was insignificant.

## 4 DISCUSSION

While the mean age of all the participants was 31.1 years, it was 30.8 years for women that was higher from the US sample (Ledikwe et al., 2007) and lower from the UK sample (Day et al., 2012). On the contrary, the mean ages of men in both the UK and the Turkish sample were quite similar to each other. While only women in the UK and the Turkish samples were in normal BMI range, women participants in the US study were overweight as were the UK and the Turkish men participants. Both women and men in the Turkish sample displayed higher scores for Cognitive Restraint of Eating; Disinhibition of Eating Control; and Susceptibility to Hunger in comparison with the US and the UK studies (Day et al., 2012; Ledikwe et al., 2007). Unexpectedly, women had lower tendency to eat emotionally than men contrary to the results of the Swedish Obese Subjects (SOS) study (Karlsson et al., 2000). This difference may result from the obese subjects enrolled in that study while our women participants were at normal BMI range. In our study, women showed lower Cognitive Restraint of Eating scores than men although this difference was statistically insignificant. In the UK sample, in contrast to our study, women had higher cognitive restraint scores. The scores of the subscale Susceptibility to Hunger for both women and men were quite similar to each other in the Turkish sample. When compared with the UK sample (study 2) (Day et al., 2012), the DIFF scores of the Turkish sample were slightly higher. Patterns were similar to that observed in both the US sample and the UK sample data (Day et al., 2012; Ledikwe et al., 2007). High‐fat foods over low‐fat alternatives were selected as eaten significantly more often among men as the same pattern observed in the UK sample (Day et al., 2012). In accordance with these results, dietary fat restriction (DIFF) scores found to be quite similar in all three different samples, which is higher among women than in men in both the UK and the Turkish sample studies. High‐fat foods that taste better (TASTE) were positively correlated with the foods eaten most often, which is similar to that reported from the UK and the United States (Day et al., 2012; Ledikwe et al., 2007). Contrary to the UK sample study (Day et al., 2012), the TASTE and the DIFF scores were found positively correlated among the Turkish sample similar to the results that Ledikwe et.al. (2007) reported in their two free‐living studies (Ledikwe et al., 2007). As the frequency of consumption of high‐fat foods increased, the dietary restriction decreased, which was reported the same for both the US and the UK samples (Day et al., 2012; Ledikwe et al., 2007). Even it was suggested that overweight and obesity were associated with increased preference for high‐fat food (Besnard, 2016; Davis et al., 2007; Douglas Braymer et al., 2017; Martínez‐Ruiz et al., 2014; Van Langeveld et al., 2018), a systematic review and meta‐analysis found no relationship between fat taste threshold or integrity and BMI (Tucker et al., 2017). Similarly, we found no relationship between the BMI and any scores of the Fat Preference Questionnaire^©^. The mean BMI value of the sample was at normal range, and participants were not grouped according to BMI. Thus, for more reliable and significant results for the suggested relationship between BMI and fat preference, a sample with participants grouped as lean; overweight; and/or obese will be helpful in further studies.

In terms of the eating inventory subscales, BMI was negatively correlated with the Emotional Eating and the Susceptibility to Hunger, which is not in accordance with the SOS study (Karlsson et al., 2000). There was a positive correlation with the DIFF scores (*r* = .14, *p* <.05) similar to the UK sample study (Day et al., 2012). However, no relationship was found with the TASTE scores (*p* >.05) in the Turkish sample as it was reported in the US sample free‐living study (Ledikwe et al., 2007). Besides, subscales of the Cognitive Restraint of Eating and the Emotional Eating did not correlate with any scores of the Fat Preference Questionnaire^©^. These results were not in an agreement with both the US and the UK sample studies, which reported that the Cognitive Restraint of Eating was negatively correlated with the TASTE but positively correlated with the DIFF scores (Day et al., 2012; Ledikwe et al., 2007). In both the UK and the Turkish sample studies, a correlation between the Susceptibility to Hunger and the FREQ was observed, but contrary to the UK results (Day et al., 2012), the correlation was negative. As it is likely to find a negative correlation between FREQ and Susceptibility to Hunger, both may induce one another; thus, it is complicated to explain the cause‐and‐effect relationship. Additionally, the Susceptibility to Hunger was positively correlated with the dietary restraint (DIFF) (*r* = −.13, *p* <.05).

The preference for high‐fat foods (FREQ) was observed to be associated with sex, which explains almost 5% of the variances in the scores of FREQ. This result indicates that women (who had a normal BMI in our sample) preferred low‐fat foods contributing to the inconsistent literature commonly focused on sex differences in overweight/obese subjects (Drewnowski & Moskowitz, 1985; Freeman et al., 2020; Karlsson et al., 2000; Van Langeveld et al., 2018).

Firstly, this study provided the first questionnaire useful for the assessment of spontaneous fat preference among Turkish population. The Turkish version of the Fat Preference Questionnaire^©^, which is developed in the United States and validated in the UK, is the first and a reliable tool for further studies regarding fat preference and fat restriction. Ledikwe et al. developed the tool among women, and Day et al. included both samples (Day et al., 2012; Ledikwe et al., 2007). Similarly, this study sample also included both sex.

Secondly, women in normal BMI range had preferences on low‐fat food differently from the previously studied overweight and obese women. Contributing to the inconsistent literature (Tucker et al., 2017), BMI was not correlated with fat preference.

Additionally, fat preference was not associated with the Cognitive Dietary Restraint that is commonly studied in similar research. While the Disinhibition of Eating Control reflects the tendency to overeat in response to the palatability of food, (Bryant et al., 2007) it did not contribute to fat preference and dietary fat restriction significantly and the Susceptibility of Hunger, indicating the sensitivity for hunger inducing excessive food intake (Bryant et al., 2007).

## 5 LIMITATIONS

As it is recommended in UK study (Day et al., 2012), we studied both sexes and found interestingly women preferred low‐fat foods, but men were not fairly represented. Additionally, the BMI classes were also not represented fairly in both women and men. Further studies are recommended to include samples grouped with different BMI classes in both sexes to clarify sex differences in different body weight statuses. Another limitation is recording body weight and height with the statement of the participants. BMI was calculated to evaluate obesity even it may not be a gold standard; thus, further studies may consider additional direct measurements.

## 6 CONCLUSION

The Turkish version of the Fat Preference Questionnaire^©^ is a statistically acceptable, valid, and reliable tool. Fat preference correlates with Disinhibition of Eating Control and Susceptibility to Hunger, while fat restriction correlates only with Disinhibition of Eating Control although none correlates with BMI; Cognitive Restraint; and Emotional Eating. As this study was conducted among a sample from an urban area of Turkey with a high educational status and mid‐upper social class, it is not a representative sample for the general Turkish population. Further studies with different representative samples are recommended.

## CONFLICT OF INTEREST

The authors declare that they have no conflict of interests. This research did not receive any specific grant from funding agencies in the public, commercial, or not‐for‐profit sectors.

## ETHICAL APPROVAL

This study was performed with the ethical approval of Marmara University of Noninvasive Researches Ethics Committee of Faculty of Health Sciences with number and date: 19.10.2018/27, conforming to the Declaration of Helsinki. A written consent form was obtained from all participants before data collection.

## TRANSPARENCY DECLARATION

The lead author affirms that this manuscript is an honest, accurate, and transparent account of the study being reported. The reporting of this work is compliant with STROBE guidelines. The lead author affirms that no important aspects of the study have been omitted and that any discrepancies from the study as planned have been explained.
